# Assessment of the knowledge of healthcare workers on monkeypox in Nigeria

**DOI:** 10.3205/dgkh000493

**Published:** 2024-08-20

**Authors:** Uche Eze, Nnenna Okafor, Gerald Ozota, Kelechi Nworie, Christian Asogwa, Ifeanyi Richard, Ann-Pearl Ilochonwu, Samuel Ezeasor, Chineye Okorie, Kenechukwu Ben-Umeh, Adaeze Ezeh, Mercy Aboh, Abdulmuminu Isah

**Affiliations:** 1 Department of Clinical Pharmacy and Pharmacy Management, University of Nigeria, Nsukka, Nigeria; 2 Department of Obstetrics and Gyneacology, Enugu State University Teaching Hospital, Enugu, Nigeria; 3 Maun Private Hospital, Maun, Botswana; 4 Department of Biochemistry, Nnamdi Azikiwe University, Awka, Anambra State, Nigeria; 5 Department of Pharmacotherapy, University of Utah, USA; 6 Department of Community Medicine, Enugu State University Teaching Hospital, Enugu, Nigeria; 7 Department of Microbiology and Biotechnology, National Institute for Pharmaceutical Research and Development, Abuja, Nigeria

**Keywords:** monkeypox, healthcare workers, knowledge, Nigeria

## Abstract

**Background:**

Monkeypox, a re-emerging zoonotic disease caused by the monkeypox virus (MPXV), poses a public health challenge in Nigeria. To effectively combat this disease, it is essential to assess the knowledge of healthcare workers (HCWs) in Nigeria concerning monkeypox outbreak.

**Methods:**

A cross-sectional web-based survey with 609 healthcare workers in Nigeria was conducted using a structured questionnaire to assess their knowledge of monkeypox. Data were coded and analyzed with Microsoft Excel and Python in Anaconda Jupyter Notebook.

**Results:**

The majority of respondents (n=318, 52.2%) had good knowledge of MPXV but also had knowledge gaps regarding certain symptoms and disease similarities. Interestingly, respondents were completely unaware of the possibility of sexual transmission of the disease. However, they recognized the possible significant impact of monkeypox on the social and economic lifestyle of Nigerians (n=582, 95.6%, adjOR=21.181, 95% CI: 14.450–31.051). Respondents had mixed knowledge regarding the use of smallpox vaccines and antiviral agents for monkeypox prevention and treatment. Furthermore, a significant proportion (n=526, 86.4%, adjOR=0.159, 95% CI: 0.126–0.201) attributed the outbreak to bioterrorism. The logistic regression highlighted a strong influence of academic qualification, type of healthcare provider, years of experience, and geopolitical zone of practice, on monkeypox knowledge in Nigeria.

**Conclusion:**

The study highlights the importance of continuous education for healthcare professionals in Nigeria to improve monkeypox outbreak management. Despite their moderate performance, there are knowledge gaps in critical areas among HCWs, necessitating further research to explore reasons and influencing factors for knowledge levels.

## Zusammenfassung

**Hintergrund:**

Affenpocken, eine wiederaufgetretende Zoonose, die durch das Affenpockenvirus (MPXV) verursacht wird, stellt eine Herausforderung für die öffentliche Gesundheit in Nigeria dar. Um diese Krankheit wirksam zu bekämpfen, ist es wichtig, das Wissen der Beschäftigten im Gesundheitswesen in Nigeria über den Ausbruch der Affenpocken zu bewerten.

**Methode:**

Anhand eines strukturierten Fragebogens wurde eine webbasierte Querschnittserhebung bei 609 Beschäftigten des Gesundheitswesens in Nigeria durchgeführt, um ihr Wissen über Affenpocken zu ermitteln. Die Daten wurden mit Microsoft Excel und Python in Anaconda Jupyter Notebook kodiert und analysiert.

**Ergebnisse:**

Die Mehrheit der Befragten (n=318, 52,2%) verfügte über gute Kenntnisse über MPXV, hatte aber Wissenslücken in Bezug auf bestimmte Symptome und Ähnlichkeiten mit anderen Krankheiten. Interessanterweise waren sich die Befragten der Möglichkeit einer sexuellen Übertragung der Krankheit überhaupt nicht bewusst. Sie erkannten jedoch die möglichen erheblichen Auswirkungen der Affenpocken auf die soziale und wirtschaftliche Lebensweise der Nigerianer (n=582, 95,6%, adjOR=21.181, 95% CI: 14.450–31.051). Die Befragten hatten unterschiedliche Kenntnisse über die Verwendung von Pockenimpfstoffen und antiviralen Mitteln zur Vorbeugung und Behandlung von Affenpocken. Außerdem führte ein signifikanter Anteil (n=526, 86,4%, adjOR=0.159, 95% CI: 0.126–0.201) den Ausbruch auf Bioterrorismus zurück. Die logistische Regression zeigte einen starken Einfluss der akademischen Qualifikation, der Art des Gesundheitsdienstleisters, der Jahre an Erfahrung und der geopolitischen Zone, in der man praktiziert, auf das Wissen über Windpocken in Nigeria.

**Schlussfolgerung:**

Die Studie unterstreicht, wie wichtig eine kontinuierliche Fortbildung des Gesundheitspersonals in Nigeria ist, um den Umgang mit dem Ausbruch der Affenpocken zu verbessern. Trotz ihrer mäßigen Fortschritte gibt es bei den Beschäftigten des Gesundheitswesens Wissenslücken in kritischen Bereichen, was weitere Untersuchungen zur den Gründen und den Einflussfaktoren auf den Wissensstand erforderlich macht.

## Introduction

Monkeypox is a re-emerging zoonotic disease caused by the monkeypox virus (MPXV), a double-stranded DNA virus in the *Orthopoxvirus* genus and Poxviridae family [1], [2]. Although monkeys and wild rodents are potential reservoirs, the specific host remains uncertain [3], [4], [5]. Human transmission occurs via respiratory, skin, or mucous membrane contact with infected animal/human droplets, fluids, or materials [6].

Following HPMX infection, there is an incubation period of 7–17 days (or 5–21 days), followed by fever, headache, fatigue, and lymphadenopathy [7]. As fever subsides, rashes appear mainly on the face, progressing through macular, papular, vesicular, and pustular phases [8]. Importantly, monkeypox's clinical presentation resembles smallpox but with milder symptoms, including lymphadenopathy, which is absent in smallpox [9], [10].

The first report of MPXV was in 1958 during an outbreak in Asian monkeys used for polio vaccine research in Denmark [11]. It was not classified as a human pathogen until 1970, when it was found in a patient in the Democratic Republic of Congo. Since then, it has become endemic in Central and West Africa [12]. In 2003, the first case outside of Africa was reported in the USA, and as of July 10, 2022, the WHO recorded 8,238 confirmed cases from 57 non-endemic countries [13]. Scientists attribute the rising cases to factors such as the end of smallpox vaccination, virus mutation, ecosystem changes, and increased human-wildlife interactions [14].

A few studies on HMPX knowledge among healthcare workers (HCWs) exist in Indonesia, Italy, and Saudi Arabia. In Indonesia, only 10% of 432 general practitioners demonstrated good knowledge [15]. Italian HCWs had significant knowledge gaps and underestimated MPXV as a pathogen [2]. In Saudi Arabia, 18.6% of 398 physicians were educated on HMPX, and many lacked knowledge on transmission, differences from other diseases, evolution, and management [16]. Similar studies should be conducted in endemic countries, including Nigeria, to provide an evidence base for policy-making and training.

Nigeria has been significantly affected by HMPX, with cases dating back to 1971. In 2017, the country experienced the world’s largest HMPX outbreak, and incidents continued in 2019, affecting multiple states [17], [18], [19]. The re-emergence of HMPX threatens global health systems, especially post-Covid-19 [20]. Lack of HMPX knowledge among HCWs is a major challenge, but limited studies have assessed it in Nigeria. This study aims to bridge this gap by assessing Nigerian HCWs knowledge of HMPX transmission and prevention.

## Materials and methods

### Study design and setting

The cross-sectional web-based study took place from October 2022 to February 2023 and included 609 HCWs from various fields and workplaces, including tertiary and general hospitals across Nigeria. It aimed to assess their knowledge regarding MPXV transmission and prevention.

### Sample size estimation

We estimated the sample size based on data from various sources [20], [21], [22], [23] and news reports [24] on the distribution of healthcare professions in Nigeria. Using the Cochran formula, we calculated a sample size of 384 participants, aiming for a 5% margin error at a 95% confidence interval.

Data collection tool: We used a semi-structured questionnaire with four parts. The first part gathered socio-demographic information. The second part included ten questions about monkeypox signs and symptoms. The third part contained 12 multiple-choice questions on monkeypox transmission, and the fourth part had six multiple-choice questions about monkeypox prevention. The questions were designed based on prior studies [1], [2], [25] and WHO information [26]. Respondents used a three-point Likert scale (true, false, don’t know) to answer.

### Pilot study

Before the main study, we conducted a pilot study involving 15 HCWs to ensure question reliability, validity, and clarity. Expert review further enhanced the questionnaire’s quality.

Reliability: Test-retest reliability was assessed, and questions with a correlation coefficient above 0.7 were included in the final questionnaire. The questionnaire items achieved a Cronbach’s alpha score of 0.786, indicating good internal consistency.

### Data collection

The survey was administered both online and in printed format, with responses collected from September 4^th^ to October 24^th^, 2022.

We used a convenience sampling method to recruit participants, without offering any monetary incentives. Invitations were sent via a Google Template and through social media platforms such as WhatsApp, Facebook, Twitter, and Telegram. Printed questionnaires were also distributed in various hospitals across Nigeria. Participants had to provide informed consent before participating and completed the survey by clicking the “submit” button. All questions had to be answered for a valid response.

### Data analysis

Data was coded in MS Excel and analyzed using Anaconda Jupyter Notebook version 5.6 [27] with Python 3.9 packages. Statistical significance was set at p<0.05. We used the Modified Alexander Govern test for statistical hypothesis tests on boxplots and visualized data with Matplotlib version 2.2 [28] and Seaborn version 0.11.2 [29]. Statsmodel package version 0.13.5 was used for logistic regression and the Pearson chi-squared test of independence [30]. The Cronbach alpha score was calculated with statistical package for social sciences (IBM SPSS statistics version 26).

To assess knowledge levels, we calculated median and mean knowledge scores from participants’ responses to 28 questions. Correct answers received a score of 1, while incorrect answers received a 0. The median score was used as a cutoff to categorize knowledge as either good or poor, due to the negatively skewed distribution of scores. Participants scoring above the median were considered to have good knowledge, while those below were categorized as having poor knowledge of monkeypox among healthcare providers in Nigeria.

### Ethical clearance

The study strictly adhered to ethical guidelines, obtaining clearance from the Institutional Ethics Committee of the University of Nigeria, Nsukka. Prior to their participation in the study, informed consent was obtained from all eligible respondents. To ensure confidentiality, the responses provided by the participants were handled with utmost discretion throughout the study period.

## Results

Table 1 (Tab. 1) provides demographic and knowledge score information for the 609 participants in the healthcare study, highlighting age, gender, education, practice settings, experience levels, geographic representation, and knowledge scores across different categories. 70.3% of the participants were 25–35 years old. The sex ratio was almost balanced. 81.8% had academic education. 74.2% were employed in hospitals and represented the group of people particularly relevant for the research question. The proportion of doctors was 32.7%, with nurses comprising 20.7%. 40.6% had a professional experience of <2 years, 43.5% of 2–5 years. The participants came from all parts of Nigeria, with different healthcare qualifications and occupations. Phyiscians and healthcare workers practicing in the Northwest region demonstrated the highest cumulative knowledge score of 21.75 and 20.85 respectively (Table 1 (Tab. 1)).

**Figure 1:**
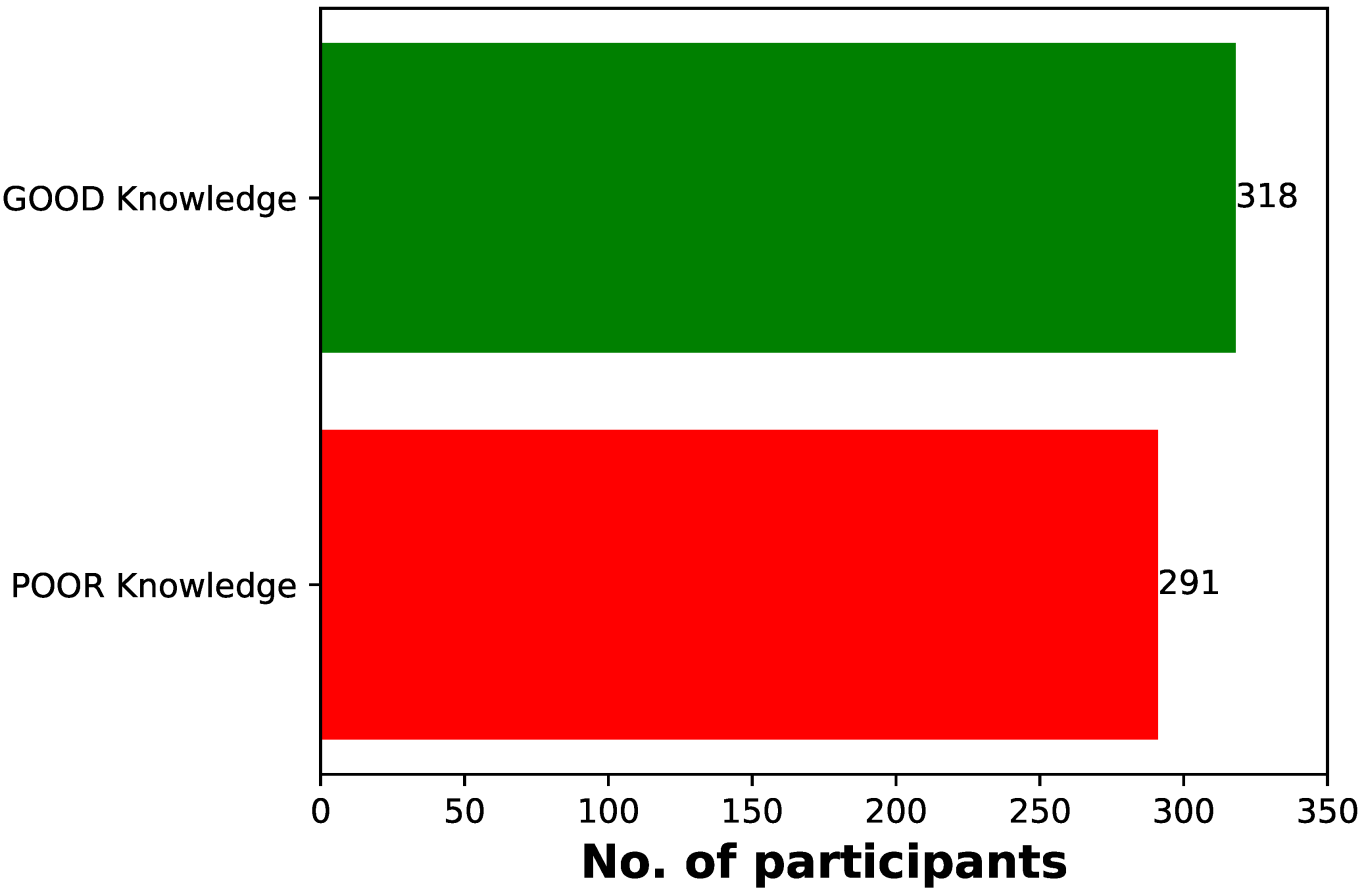
Bar chart analysis of the cumulative knowledge levels

The majority of respondents (n=318, 52.2%) had a good knowledge of MPXV, as they were able to identify the symptoms, risk factors and prevention of the virus, while (n=291, 47.8%) had poor knowledge of MPXV (Figure 1 (Fig. 1)).

**Figure 2:**
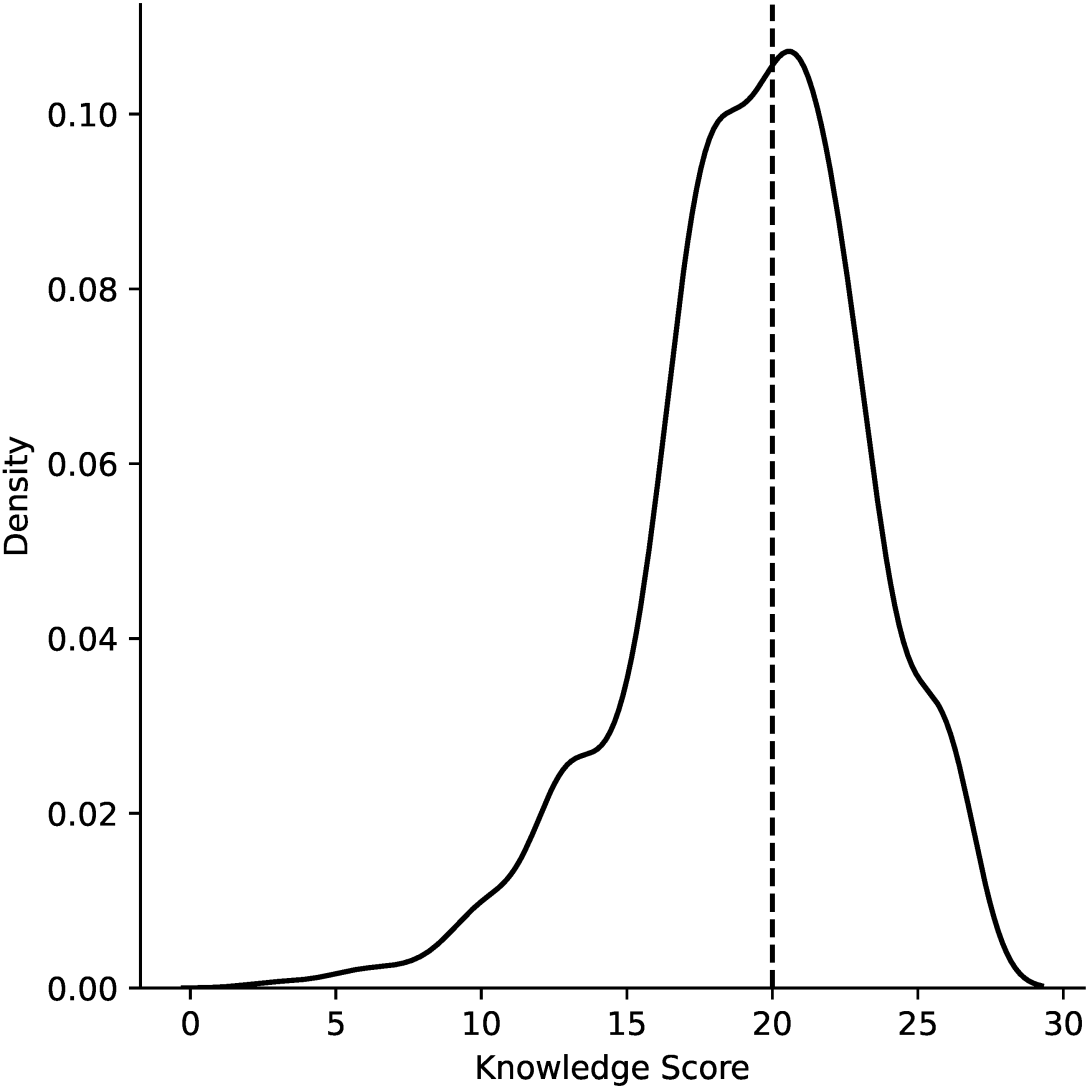
Knowledge score distribution

The distribution of knowledge scores, as illustrated in Figure 2 (Fig. 2), exhibits characteristics of a normal distribution, but with a notable negative skew. This skewness is evidenced by a concentration of values towards the right side of the plot, indicating that a majority of participants possessed a strong understanding of the subject matter. However, it is essential to acknowledge the presence of outliers with lower scores, representing a subgroup of respondents who demonstrated a weaker grasp of the topic. The mean knowledge score was calculated at 19.3 (±3.9), which is notably less than the median score of 20.0 (±5.0). This discrepancy between the mean and median underscores the skewed nature of the distribution. As a result, utilizing the median score as a measure of central tendency for the knowledge score is justified (Figure 2 (Fig. 2)).

**Figure 3:**
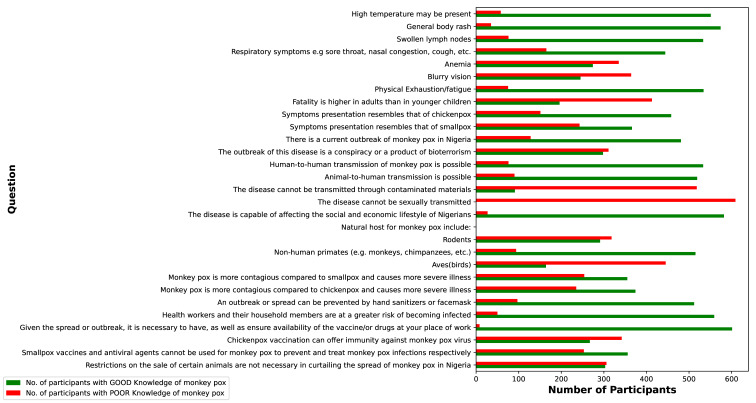
Bar chart analysis assessing knowledge levels on monkeypox symptoms, transmission, and prevention among respondent

Figure 3 (Fig. 3) presents bar charts categorizing respondents into two groups: those with good and poor levels of knowledge for each study question. The results indicate that the majority of respondents demonstrated a strong grasp of monkeypox symptoms, transmission, and prevention. However, there was a notable gap in understanding regarding the disease’s natural host and its potential transmission through contact with contaminated surfaces.

**Figure 4:**
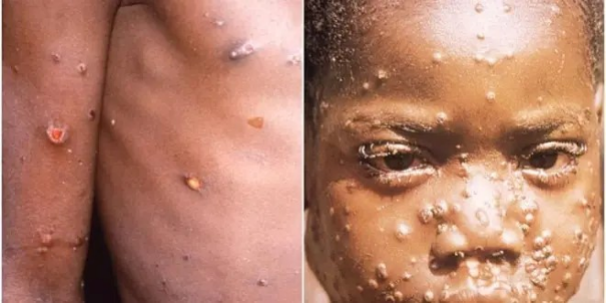
Image of a patient with typical vesiculopustular monkeypox symptoms (adapted from [62])

Figure 4 (Fig. 4) shows images from a news article reporting on the World Health Organization’s (WHO) confirmation of 3,200 monkeypox cases across 48 countries, including Nigeria [31]. It provides insights into the global impact of monkeypox outbreaks and can be a valuable resource for understanding the disease’s reach.

**Figure 5:**
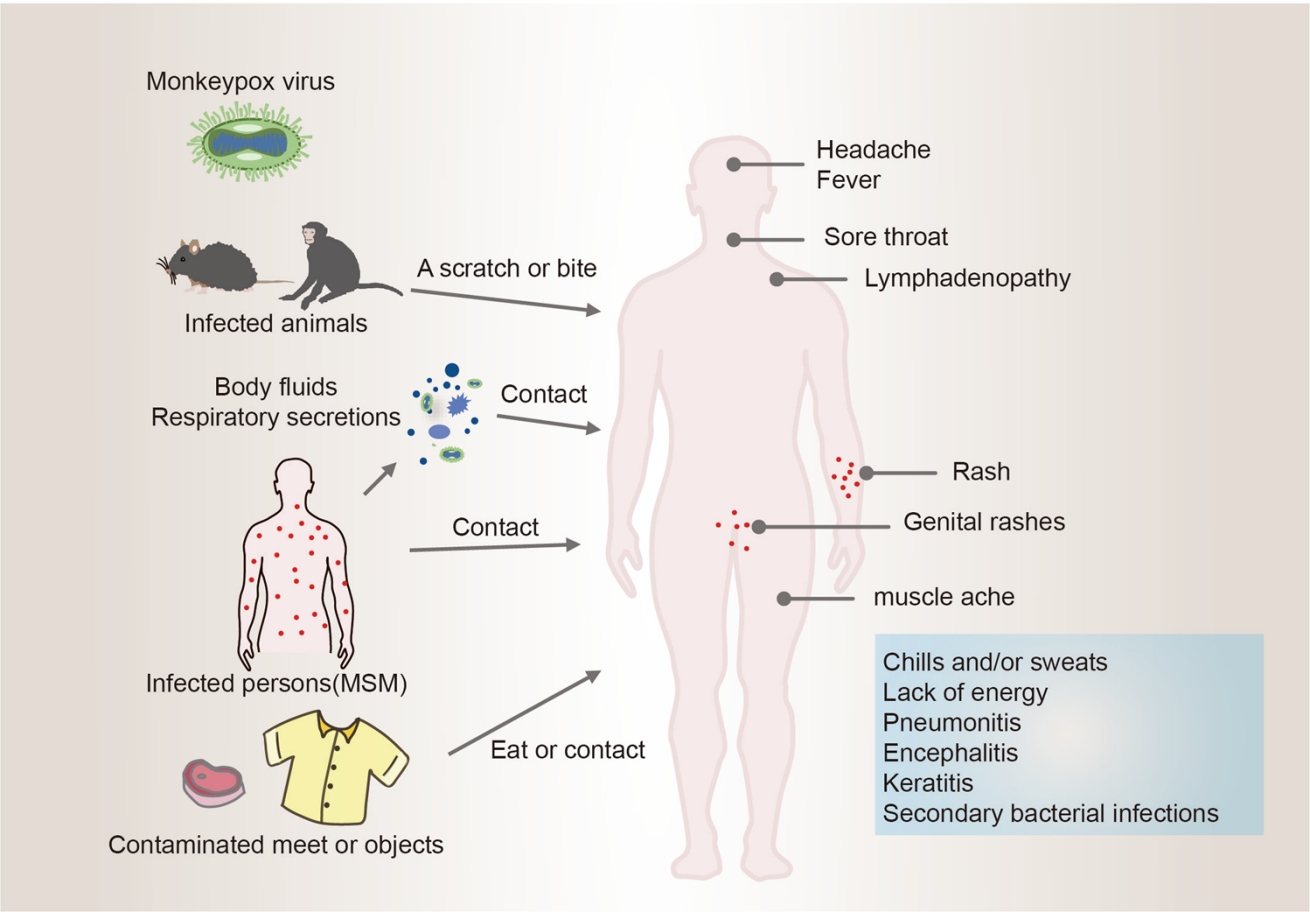
Transmission routes and clinical characteristics of monkeypox (adapted from [61])

The virus can spread through various means, e.g., contact with infected animals, meat consumption, sexual contact (especially among men who have sex with men) and exposure to respiratory secretions. Key symptoms, such as elevated temperature, headaches, skin and genital eruptions, are depicted, along with possible complications like pneumonitis (lung inflammation ) encephalitis (brain inflammation), keratitis (eye infections), and bacterial superinfections (Figure 5 (Fig. 5), [32]).

**Figure 6:**
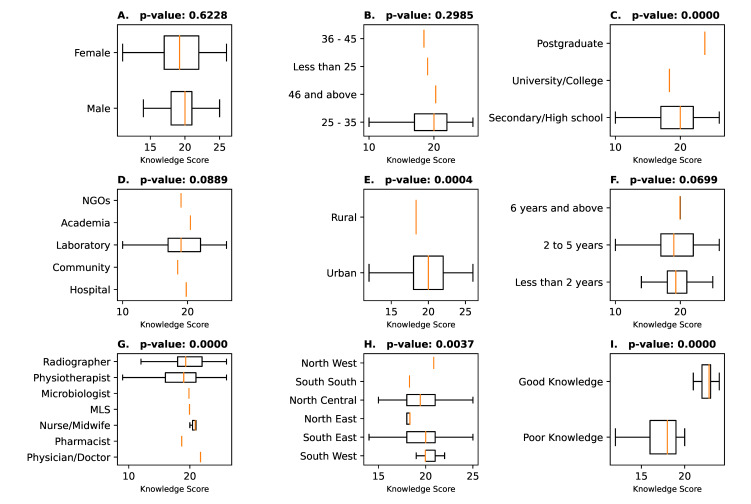
Boxplots of the variability/spread of knowledge scores among the socio-demographic variables

Figure 6 (Fig. 6) displays box plots illustrating knowledge scores (KS) in the context of socio-demographic factors. Notably, p-values <0.005 indicate highlight significant disparities in knowledge. Bar plots without boxes imply limited variability, indicating a lack of knowledge gaps. The box height signifies central tendency, and the box length represents knowledge spread, with wider boxes indicating greater variability. In general, when considering socio-demographic groups, only two variables (age and area of practice) exhibited low variability across the factors, except for the 25–35 age group (KS=19.46) and those in laboratory practice (KS=19.7). Furthermore, several variables, such as physicians/doctors (KS=21.75), respondents from the Northwest region (KS=20.85), healthcare workers in academia (KS=20.44), nurse/midwife practitioners (KS=20.88), and respondents aged above 45 years (KS=20.27) surpassed the median score, signifying a high level of knowledge with minimal variability, as evidenced by the tight distribution of their boxplots (Figure 6 (Fig. 6)).

Table 2 (Tab. 2) presents participants’ knowledge of monkeypox, including percentages, chi-squared test results, adjusted odds ratios (adjOR), and confidence intervals. Respondents demonstrated good awareness of common monkeypox symptoms but showed limited knowledge of less common symptoms. A substantial proportion (97.4%) believed vaccines/drugs should be available at their workplaces (adjOR=35.969, 95% CI: 22.054–58.666). Additionally, respondents recognized the heightened risk of infection among healthcare workers and their households (98.7%). About 51.6% of respondents agrees that the outbreak resulted from bioterrorism or conspiracy theories. Notably, respondents were unaware of sexual transmission possibilities (adjOR=0.001, 95% CI: 0.000–0.013). Respondents overwhelming (95.6%) opined that the outbreak can affect the socio-economic realities in Nigeria. Opinions on the necessity of restricting animal sales to control monkeypox varied (50.3% deemed it unnecessary). Respondents had somewhat mixed knowledge on the use of chickenpox vaccines with good knowledge at (43.9%), and poor knowledge at (56.1%). Knowledge regarding smallpox vaccines and antiviral agents for monkeypox prevention and treatment was mixed, with many believing they cannot be used (58.5%, adjOR=1.406, 95% CI: 1.197–1.65) (Table 2 (Tab. 2)).

Table 3 (Tab. 3) presents the binomial logistic regression analysis indicating the impact of various socio-demographic factors on the level of knowledge regarding monkeypox in Nigeria and identifies key determinants of knowledge in healthcare workers. From the logistic regression results, age (p-value=0.233), gender (p-value=0.326), academic qualification (p-value=0.197), area of practice (p-value=0.076), and location of practice (p-value=0.179) have no effect on the knowledge of monkeypox in Nigeria, which signifies that these factors are not associated with differences in knowledge levels among respondents. The findings also reveal academic qualification (p-value=0.000), type of healthcare provider (p-value=0.011), years of experience (p-value=0.008) and geopolitical zone of practice (p-value=0.000) to have a strong influence or impact on the knowledge levels of HCWs about monkeypox in Nigeria.

## Discussion

The WHO has outlined key areas for addressing the current HMPX outbreak, emphasizing emergency coordination, surveillance, community protection, care, and research [33]. HCWs’ timely and appropriate responses are essential. To achieve this, it is crucial to evaluate HCWs’ initial knowledge of HMPX, especially among physicians and nurses who play a vital role in patient care. This assessment can enhance awareness and support informed training, aligning with WHO recommendations [34]. Monkeypox, a zoonotic disease caused by the monkeypox virus, demands proficient healthcare practitioners for effective management [26]. Hence, educating healthcare professionals about monkeypox is vital due to its high transmissibility [35], especially in light of recent outbreaks in Africa with numerous cases and fatalities [12]. Respondents recognized the heightened risk of infection among healthcare workers and their households (98.7%). Given that healthcare workers are often the first point of contact for patients, addressing this knowledge gap among them is particularly critical.

From our study, Nigerian HCWs demonstrated good knowledge of the monkeypox virus (52.2%), successfully identifying clinical presentation and transmission modes. A significant number of participants in this study (87.5%) had a good understanding that human-to-human transmission is possible, which agrees with findings from Saudi Arabia by Temsah et al. [36]. A study conducted by Alshahrani et al. [37] revealed that although a substantial portion of the study participants exhibited a sound comprehension of human-to-human transmission, the majority still struggled to accurately identify that a bite from infected monkeys could also serve as a mode of virus transmission. The findings of the present study may be attributed to extensive sensitization efforts by the Nigeria Center for Disease Control (NCDC), including prevention guidelines and management protocols [38]. Previous studies reported a lower proportion of correct responses regarding human-to-human transmission [1], [25], [39]. Studies among HCWs in Jordan reported poor MPXV knowledge, with study participants scoring above 70% in only four out of eleven HMPX knowledge questions [39]. A study in the Czech Republic also revealed suboptimal knowledge, possibly linked to lower outbreak frequency or endemicity [40]. It is important to note that person-to-person transmission of the monkeypox virus has been observed in the past, and it became more apparent during the recent outbreak in 2022. However, close contact is necessary for transmission, and infection does not occur as easily as with respiratory viruses [41], [42]. Respondents in this study displayed a total lack of awareness regarding the possibility of sexual transmission of monkeypox, despite scientific evidence to the contrary [39], [43], [44], [45], [46]. In order to mitigate the global impact of monkeypox, a reduction in number of sexual partners could potentially slow down the transmission of the disease, leading to a decrease in the number of infected individuals [47]. Further research and surveillance are needed to clarify this issue, as it could have significant implications for public health and disease prevention measures. 

Notably, our study highlights the relationship between socio-demographics and knowledge about MPXV among HCWs, as demonstrated by the binomial (binary) logistic regression analysis. It suggests that factors such as higher academic qualifications, type of healthcare provider, years of experience, and geopolitical location of practice are significantly correlated with knowledge scores on monkeypox in Nigeria. However, it is important to note that correlation does not imply causation, and more research may be needed to fully understand these patterns. To effectively control monkeypox in Nigeria, future strategies should consider these factors and tailor training to address knowledge gaps among healthcare professionals.

Our logistic regression revealed type of healthcare provider as a predictor variable for knowledge of monkeypox outbreak in Nigeria. Impressively, physicians demonstrated a greater understanding of the condition as a subgroup of type of healthcare providers, with an above median score of 21.75. This finding agrees with previous studies in Kuwait and Jordan, which indicated that physicians had higher levels of knowledge [1], [39]. The third best performing subgroup after physicians and nurses/midwives were medical laboratory scientists who also produced an above median score of 19.94. It is important to highlight this finding as medical laboratories play crucial roles in detecting monkeypox cases, and ensuring effective responses and preparedness [48]. Their vital role in combating outbreaks could account for why medical laboratory scientists seem to have a greater awareness about the disease when compared with the other professions that participated in this study other than physicians and nurses. Our study encompassed various sectors of the Nigerian healthcare workforce, which can shed light on the roles of different healthcare professionals in delivering comprehensive care during outbreaks. This underscores the pivotal importance of a robust healthcare infrastructure and preparedness for effectively managing infectious disease outbreaks [49]. In effect, a multidisciplinary approach involving healthcare professionals, policymakers, and community engagement emerges as an imperative strategy for preventing and managing monkeypox outbreaks [50].

Furthermore, our logistic regression also revealed the significant relationship between years of experience as a healthcare worker and knowledge scores, bringing to light the important role of experience in diagnosing and managing monkeypox. A study in Jordan revealed that a considerable portion of the participants expressed lower confidence in their capacity to handle and identify cases of Human Monkeypox (HMPX), which was related to their existing knowledge and skill level [39]. In addition to various contributing factors, such as inadequate training and limited information access, our findings highlight the significance of years spent in clinical practice. It is evident that the duration of practice impacts HCWs’ confidence levels in monkeypox diagnosis [39], [51]. Moreover, information accessibility emerges as a potent determinant of comprehension, even among HCWs with limited experience [52]. Notably, the sources of information wield substantial influence over knowledge levels, as corroborated by a previous study in Nigeria [53]. While our investigation revealed commendable overall knowledge, some respondents exhibited knowledge gaps in comprehending transmission modes, which can result from from unreliable sources of information, such as social media. A study conducted by Harapan et al. [15] among general practitioners in Indonesia revealed that a majority of them receive their information from online media. Therefore, emphasizing the prevalence of misinformation and deliberate disinformation becomes paramount when discussing the emergence of infectious diseases [54]. A worrying revelation from our study, echoing the influence of misinformation often found on social media platforms, is that a proportion of respondents (51.6%) linked the outbreak to bioterrorism. This predisposition toward conspiracy theories is often fueled by lower knowledge levels, which can perpetuate unfounded beliefs regarding bioterrorism as the outbreak’s root cause [25]. This susceptibility to conspiracy theories was notably associated with an inadequate understanding of the human monkeypox virus [1]. However, another study found that the respondents generally held a neutral stance toward conspiracy theories related to emerging virus infections [39]. To bridge these knowledge gaps, comprehensive educational initiatives are imperative, with a focus on accurate information dissemination. Continuous professional development programs and webinars conducted during outbreaks serve as essential tools for keeping HCWs well-informed. While social media offer a valuable information conduit, skepticism is warranted, due to the rampant circulation of misinformation and conspiracy theories [55]. Engaging in a training program focused on the monkeypox virus was associated with a higher level of knowledge, as compared to individuals who did not participate in such training [16], [43]. Therefore, it is crucial to scrutinize the impact of experience on knowledge levels, which also ensures that HCWs remain up-to-date and self-assured in diagnosing infectious diseases. 

Variations in knowledge scores among distinct geopolitical regions emphasize regional disparities in monkeypox awareness. Notably, the North West region demonstrated the highest knowledge scores (20.85), consistent with previous findings in Nigeria [53], which also identified the Northwest region as having the highest knowledge among healthcare workers based on their geopolitical zone of practice. However, conflicting studies have suggested that regional residence insignificantly influences knowledge [56]. While the underlying reasons for these disparities remain elusive, an in-depth examination of educational policies and public health strategies in high-knowledge regions could offer valuable insights for regions lagging behind.

Also, our study featured well-educated participants, with a majority having attained university or college-level education. Notably, our findings support a substantial correlation between higher academic qualifications and knowledge scores, consistent with observations in Pakistan [57]. This association prompts intriguing inquiries into the role of education in augmenting knowledge about monkeypox. Health practitioners who completed their education at universities situated in more urbanized regions exhibited knowledge of monkeypox [16]. 

Vaccination is crucial for disease prevention, with potential economic and social benefits. Most participants in this study expressed the need for vaccines/drugs at their workplaces (97.4%). Policymakers should consider various aspects, including health, economics, and social impact, when developing vaccination policies and strategies [58]. Our data highlights a significant knowledge gap among respondents, with a substantial percentage (58.5%) expressing beliefs that smallpox vaccines and antiviral agents cannot be employed in the prevention and treatment of monkeypox. In a study conducted by Temsah et al. involving 1,130 healthcare workers in Saudi Arabia, a comparable situation arose. Their research disclosed that only a minority of participants could identify the benefits of the smallpox vaccine [36]. Similarly, our study revealed contrasting opinions on the effectiveness of chickenpox vaccination against monkeypox, with only 43.9% erroneously believing it imparts immunity. Interestingly, another parallel scenario unfolded, where a substantial majority erroneously asserted that the chickenpox vaccine, designed for chickenpox, is effective against monkeypox (MPOX) [36].

These findings underscore a substantial lack of awareness about the potential use of these preventive and therapeutic measures. The misconception regarding the ineffectiveness of smallpox vaccines and antiviral agents is concerning, as it can hinder effective monkeypox management. It is crucial to recognize that smallpox vaccines, while not specifically designed for monkeypox, have shown some degree of cross-protection due to the similarity between the two viruses. Similarly, antiviral agents can play a vital role in treating monkeypox cases, especially in severe instances. Therefore, dispelling such misconceptions and disseminating accurate information about the utility of these measures is imperative.

Nearly all participants (95.6%) recognized the significant impact of monkeypox on the social and economic lifestyle of Nigerians. The monkeypox outbreak has profound socio-economic implications, particularly for vulnerable populations. Similar to the COVID-19 pandemic, it can negatively affect public finances and exacerbate gender inequality, with women facing caregiving responsibilities and economic instability [59]. The government's initial lack of response to the monkeypox epidemic led to panic and disruption of community life in Nigeria [60]. Interventions should consider local social contexts to effectively target at-risk populations [61]. Collaboration between social workers and public health workers in creating awareness and support for affected individuals and families is essential. Increasing investment in poverty reduction and social protection programs, as well as improving access to healthcare and sanitation, can address socio-economic issues arising from the outbreak, especially in countries with an overall weak socio-economic standing [60], [62]. The outbreak can strain the health workforce and supply chains, in addition to impacting national economies, particularly in countries with a weak socio-economic status [59].

## Conclusion

The monkeypox outbreak is a complex and ever-changing situation that deeply affects society and the economy. Our study's results emphasize the importance of continuous education and awareness programs for healthcare professionals in Nigeria. These efforts aim to improve their knowledge, preparedness, and response capabilities in effectively dealing with monkeypox outbreaks. Recognizing the specific vulnerabilities of at-risk populations, customizing interventions accordingly, and promoting collaboration between social workers and public health experts are essential for reducing the adverse effects of the outbreak and establishing a stronger and fairer future. Embracing a collaborative and interdisciplinary approach enables healthcare systems to better prevent and manage the impact of monkeypox outbreaks, protecting the public and ensuring their well-being.

## Notes

### Competing interests

The authors declare that they have no competing interests.

### ORCID of the author

Uche Eze: 0009-0002-8702-4357

## Figures and Tables

**Table 1:**
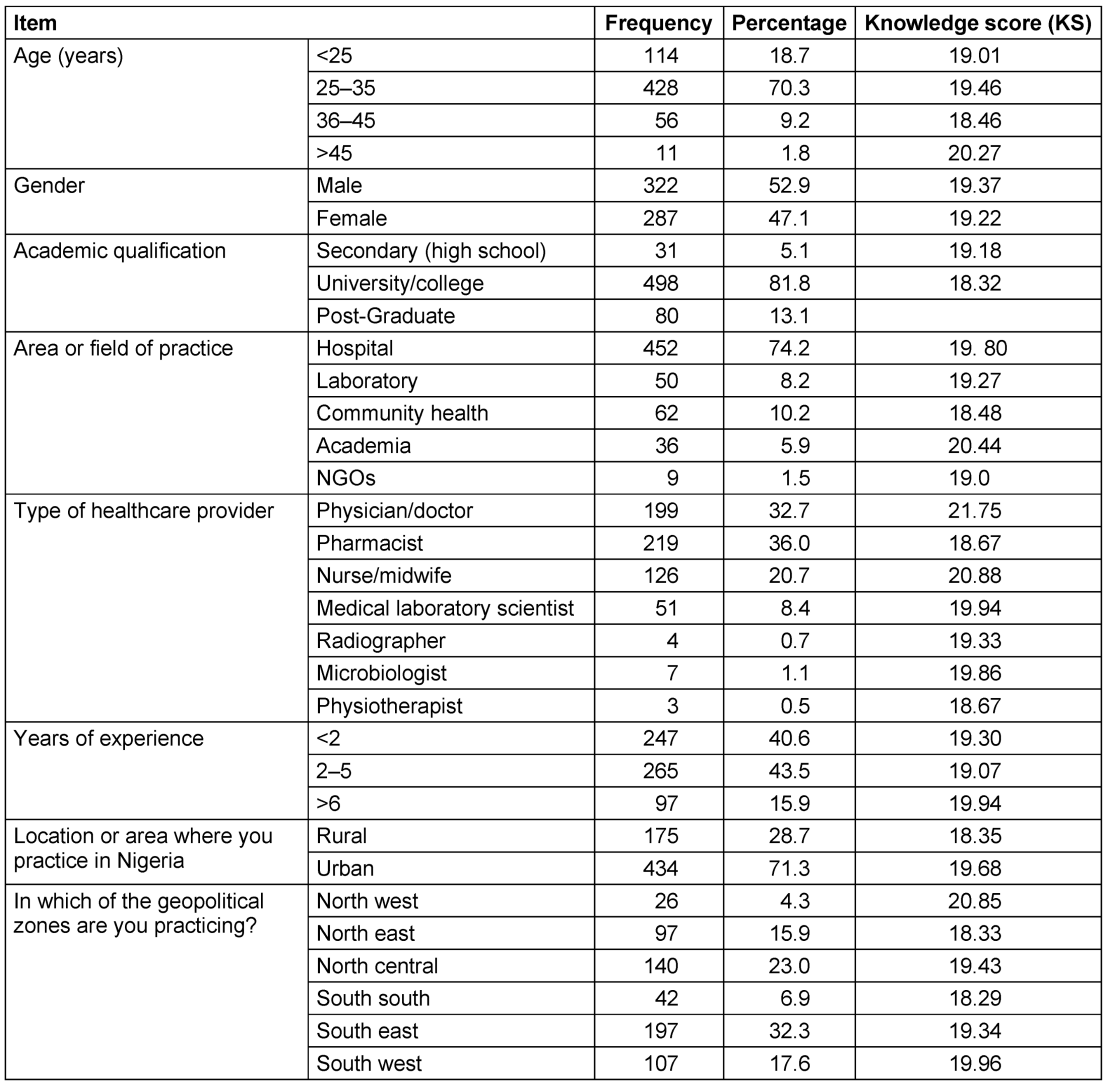
Socio-demographic characteristics of the study participants (n=609)

**Table 2:**
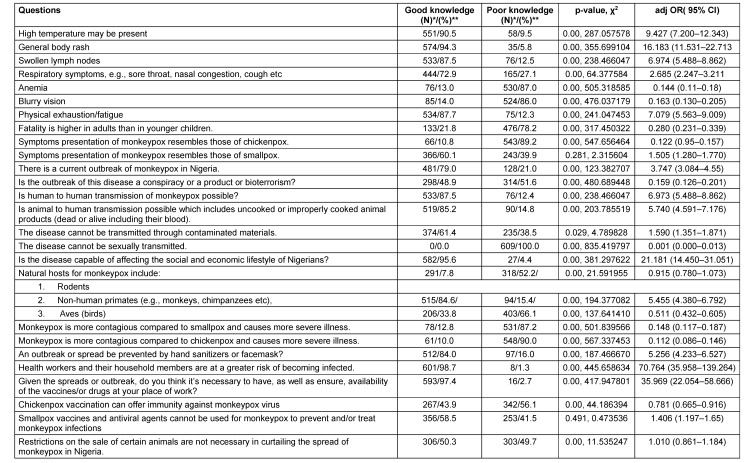
Knowledge of monkeypox symptoms and associated factors among study participants

**Table 3:**
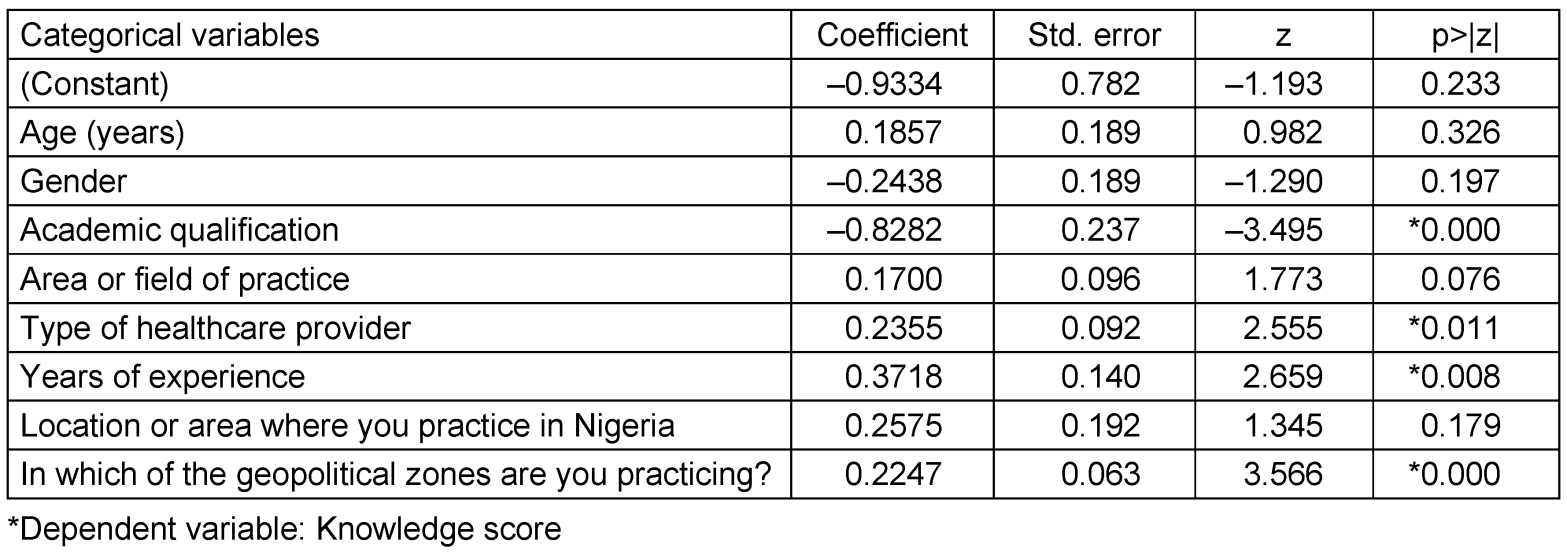
Binomial logistic regression analysis of the impact of socio-demographic factors on knowledge of monkeypox among healthcare workers in Nigeria

## References

[1] Alshahrani NZ, Alzahrani F, Alarifi AM, Algethami MR, Alhumam MN, Ayied HAM, Awan AZ, Almutairi AF, Bamakhrama SA, Almushari BS, Sah R (2022). Assessment of Knowledge of Monkeypox Viral Infection among the General Population in Saudi Arabia. Pathogens.

[2] Riccò M, Ferraro P, Camisa V, Satta E, Zaniboni A, Ranzieri S, Baldassarre A, Zaffina S, Marchesi F (2022). When a Neglected Tropical Disease Goes Global: Knowledge, Attitudes and Practices of Italian Physicians towards Monkeypox, Preliminary Results. Trop Med Infect Dis.

[3] Centers for Disease Control and Prevention (CDC) (2022). Technical Report 4: Multi-National Mpox Outbreak, United States, 2022.

[4] Petersen E, Kantele A, Koopmans M, Asogun D, Yinka-Ogunleye A, Ihekweazu C, Zumla A (2019). Human Monkeypox: Epidemiologic and Clinical Characteristics, Diagnosis, and Prevention. Infect Dis Clin North Am.

[5] World Health Organization (WHO) (2023). Mpox (monkeypox) [Factsheet].

[6] Durski KN, McCollum AM, Nakazawa Y, Petersen BW, Reynolds MG, Briand S, Djingarey MH, Olson V, Damon IK, Khalakdina A (2018). Emergence of Monkeypox - West and Central Africa, 1970-2017. MMWR Morb Mortal Wkly Rep.

[7] Centers for Disease Control and Prevention (CDC) Mpox. Signs and Symptoms.

[8] McCollum AM, Damon IK (2014). Human monkeypox. Clin Infect Dis.

[9] Damon IK (2011). Status of human monkeypox: clinical disease, epidemiology and research. Vaccine.

[10] Nalca A, Rimoin AW, Bavari S, Whitehouse CA (2005). Reemergence of monkeypox: prevalence, diagnostics, and countermeasures. Clin Infect Dis.

[11] Parker S, Buller RM (2013). A review of experimental and natural infections of animals with monkeypox virus between 1958 and 2012. Future Virol.

[12] World Health Organization (WHO) (2022). Multi-country monkeypox outbreak in non-endemic countries. Disease Outbreak News.

[13] Jamil H, Tariq W, Tahir MJ, Mahfooz RS, Asghar MS, Ahmed A (2022). Human monkeypox expansion from the endemic to non-endemic regions: Control measures. Ann Med Surg (Lond).

[14] Di Giulio DB, Eckburg PB (2004). Human monkeypox: an emerging zoonosis. Lancet Infect Dis.

[15] Harapan H, Setiawan AM, Yufika A, Anwar S, Wahyuni S, Asrizal FW, Sufri MR, Putra RP, Wijayanti NP, Salwiyadi S, Maulana R, Khusna A, Nusrina I, Shidiq M, Fitriani D, Muharrir M, Husna CA, Yusri F, Maulana R, Andalas M, Wagner AL, Mudatsir M (2020). Knowledge of human monkeypox viral infection among general practitioners: a cross-sectional study in Indonesia. Pathog Glob Health.

[16] Alshahrani NZ, Algethami MR, Alarifi AM, Alzahrani F, Alshehri EA, Alshehri AM, Sheerah HA, Abdelaal A, Sah R, Rodriguez-Morales AJ (2022). Knowledge and Attitude Regarding Monkeypox Virus among Physicians in Saudi Arabia: A Cross-Sectional Study. Vaccines (Basel).

[17] Alakunle E, Moens U, Nchinda G, Okeke MI (2020). Monkeypox Virus in Nigeria: Infection Biology, Epidemiology, and Evolution. Viruses.

[18] World Health Organization (WHO) (2018). Monkeypox. Current status in West and Central Africa. Report of a WHO informal consultation, Geneva, Switzerland, 3 November 2017. WHO/WHE/IHM/2018.3.

[19] Chieloka O, Amao L, Akinrogbe J, Iniobong JI, Burga J (2020). Outbreak investigation of monkeypox in Akwa Ibom State: a matched case control study 14th - 24th October 2019. East Afr J Health Sci.

[20] Nwankwo ONO, Ugwu CI, Nwankwo GI, Akpoke MA, Anyigor C, Obi-Nwankwo U, Andrew S, Nwogu K, Spicer N (2022). A qualitative inquiry of rural-urban inequalities in the distribution and retention of healthcare workers in southern Nigeria. PLoS One.

[21] Ekpenyong A, Udoh A, Kpokiri E, Bates I (2018). An analysis of pharmacy workforce capacity in Nigeria. J Pharm Policy Pract.

[22] Balogun JA (2020). The path to our destiny: The transitioning of physiotherapy in Nigeria from occupation to a true profession. J Nig Soc Physiother.

[23] Adejoh T (2019). An inquest into the quests and conquests of the radiography profession in Nigeria. Journal of Radiography and Radiation Sciences.

[24] Folorunsho-Francis A (2020). Unemployed laboratory scientists in Nigeria now over 30,000 -Association. Punch Healthwise.

[25] Sallam M, Al-Mahzoum K, Dardas LA, Al-Tammemi AB, Al-Majali L, Al-Naimat H, Jardaneh L, AlHadidi F, Al-Salahat K, Al-Ajlouni E, AlHadidi NM, Bakri FG, Mahafzah A, Harapan H (2022). Knowledge of Human Monkeypox and Its Relation to Conspiracy Beliefs among Students in Jordanian Health Schools: Filling the Knowledge Gap on Emerging Zoonotic Viruses. Medicina (Kaunas).

[26] World Health Organization (WHO) (2023). Questions and answers. Mpox (monkeypox).

[27] (2020). Anaconda Software Distribution, Anaconda Inc. [Internet].

[28] Hunter JD (2007). Matplotlib: A 2D graphics environment. Comput Sci Eng.

[29] Waskom ML (2021). Seaborn: statistical data visualization. J Open Source Softw.

[30] Seabold S, Perktold J, van der Walt S, Millman J Statsmodels: Econometric and statistical modeling with python.

[31] Ologunagba C (2022). WHO confirms 3,200 cases of monkeypox in 48 countries including Nigeria. Global Patriot News.

[32] Huang Y, Mu L, Wang W (2022). Monkeypox: epidemiology, pathogenesis, treatment and prevention. Signal Transduct Target Ther.

[33] World Health Organization (WHO) (2022). Second Meeting of the International Health Regulations (2005) (IHR) Emergency Committee Regarding the Multi-Country Outbreak of Monkeypox.

[34] World Health Organization (WHO) (2022). Monkeypox, COVID-19 & Other Global Health Issues Virtual Press Conference - 22 September 2022. Press briefing transcript.

[35] Titanji BK, Tegomoh B, Nematollahi S, Konomos M, Kulkarni PA (2022). Monkeypox: A Contemporary Review for Healthcare Professionals. Open Forum Infect Dis.

[36] Temsah MH, Aljamaan F, Alenezi S, Abouammoh N, Alhasan K, Dasuqi SA, Alhaboob A, Hamad MA, Halwani R, Alrabiaah A, Alsubaie S, Alshahrani FS, AlZamil F, Memish ZA, Barry M, Al-Tawfiq JA (2022). Monkeypox Disease (MPOX) Perceptions among Healthcare Workers versus General Population during the First Month of the WHO Alert: Cross-Sectional Survey in Saudi Arabia. Vaccines (Basel).

[37] Alshahrani NZ, Mitra S, Alkuwaiti AA, Alhumam MN, Altmimi SMB, Alamri MHM, Albalawi ZAS, Almorgi MW, Alharbi HKD, Alshahrani SM (2022). Medical students’ perception regarding the re-emerging monkeypox virus: an institution-based cross-sectional study from Saudi Arabia. Cureus.

[38] Ogoina D, Izibewule JH, Ogunleye A, Ederiane E, Anebonam U, Neni A, Oyeyemi A, Etebu EN, Ihekweazu C (2019). The 2017 human monkeypox outbreak in Nigeria-Report of outbreak experience and response in the Niger Delta University Teaching Hospital, Bayelsa State, Nigeria. PLoS One.

[39] Sallam M, Al-Mahzoum K, Al-Tammemi AB, Alkurtas M, Mirzaei F, Kareem N, Al-Naimat H, Jardaneh L, Al-Majali L, AlHadidi A, Al-Salahat K, Al-Ajlouni E, AlHadidi NM, Bakri FG, Harapan H, Mahafzah A (2022). Assessing Healthcare Workers’ Knowledge and Their Confidence in the Diagnosis and Management of Human Monkeypox: A Cross-Sectional Study in a Middle Eastern Country. Healthcare (Basel).

[40] Riad A, Drobov A, Rozmarinová J, Drapáčová P, Klugarová J, Dušek L, Pokorná A, Klugar M (2022). Monkeypox Knowledge and Vaccine Hesitancy of Czech Healthcare Workers: A Health Belief Model (HBM)-Based Study. Vaccines (Basel).

[41] Ciccozzi M, Petrosillo N (2022). The Monkeypox Pandemic as a Worldwide Emergence: Much Ado?. Infect Dis Rep.

[42] Kozlov M (2022). Monkeypox goes global: why scientists are on alert. Nature.

[43] Alshahrani NZ, Mitra S, Alkuwaiti AA, Alhumam MN, Altmimi SMB, Alamri MHM, Albalawi ZAS, Almorgi MW, Alharbi HKD, Alshahrani SM (2022). Medical students’ perception regarding the re-emerging monkeypox virus: an institution-based cross-sectional study from Saudi Arabia. Cureus.

[44] Salim NA, Septadina IS, Permata M, Hudari H (2022). Knowledge, attitude, and perception of anticipating 2022 global human monkeypox infection among internal medicine residents at Palembang Indonesia: an Online Survey. J Kedokt Kesehatan: Publikasi Ilmiah Fakultas Kedokteran Universitas Sriwijaya.

[45] Brundu M, Marinello S, Scaglione V, Ferrari A, Franchin E, Mazzitelli M, Cattelan AM (2023). The first case of monkeypox virus and acute HIV infection: Should we consider monkeypox a new possible sexually transmitted infection? J Dermatol.

[46] Rizzo A, Pozza G, Salari F, Giacomelli A, Mileto D, Cossu MV, Mancon A, Gagliardi G, Micol B, Micheli V, Capetti A, Antinori S, Gismondo MR, Olivieri P, Lombardi A (2023). Concomitant diagnosis of sexually transmitted infections and human monkeypox in patients attending a sexual health clinic in Milan, Italy. J Med Virol.

[47] Spicknall IH, Pollock ED, Clay PA, Oster AM, Charniga K, Masters N, Nakazawa YJ, Rainisch G, Gundlapalli AV, Gift TL (2022). Modeling the Impact of Sexual Networks in the Transmission of Monkeypox virus Among Gay, Bisexual, and Other Men Who Have Sex with Men - United States, 2022. MMWR Morb Mortal Wkly Rep.

[48] Beale S (2022). Medical Laboratories Respond to Monkeypox outbreak using CDC-developed diagnostic test.

[49] Lo Piccolo AJ, Wallach A, McPherson TD, Mgbako O, Fagan I, Pitts RA, Klinger A, Foote M, Garcia EA, Zucker JE, Chan J, Bails DB, Cohen GM, Tennill PA, Wong M, Mukherjee V (2023). The Role of a Tertiary Level Safety Net Hospital in New York City’s 2022 Mpox Outbreak. Health Secur.

[50] Amer F, Khalil HES, Elahmady M, ElBadawy NE, Zahran WA, Abdelnasser M, Rodríguez-Morales AJ, Wegdan AA, Tash RME (2023). Mpox: Risks and approaches to prevention. J Infect Public Health.

[51] Harapan H, Setiawan AM, Yufika A, Anwar S, Wahyuni S, Asrizal FW, Sufri MR, Putra RP, Wijayanti NP, Salwiyadi S, Maulana R, Khusna A, Nusrina I, Shidiq M, Fitriani D, Muharrir M, Husna CA, Yusri F, Maulana R, Utomo PS, Andalas M, Wagner AL, Mudatsir M (2020). Confidence in managing human monkeypox cases in Asia: A cross-sectional survey among general practitioners in Indonesia. Acta Trop.

[52] Miraglia Del Giudice G, Della Polla G, Folcarelli L, Napoli A, Angelillo IF, Collaborative Working Group (2023). Knowledge and attitudes of health care workers about monkeypox virus infection in Southern Italy. Front Public Health.

[53] Awoyomi OJ, Njoga EO, Jaja IF, Oyeleye FA, Awoyomi PO, Ibrahim MA, Saulawa MA, Galadima HB, Rowaiye AB, Olasoju TI, Idrisa JA, Olalere FDH, Olasoju MI, Adisa OH, Adetunji VE, Idemudia OO, Ezenduka EV, Oguttu JW (2023). Mpox in Nigeria: Perceptions and knowledge of the disease among critical stakeholders-Global public health consequences. PLoS One.

[54] Larson HJ (2018). The biggest pandemic risk? Viral misinformation. Nature.

[55] Bridgman A, Merkley E, Zhilin O, Loewen PJ, Owen T, Ruths D (2021). Infodemic pathways: evaluating the role that traditional and social media play in cross-national information transfer. Front Polit Sci.

[56] Al-Mustapha AI, Ogundijo OA, Sikiru NA, Kolawole B, Oyewo M, El-Nadi H, Mustapha AM, Adebudo LI, Odukoya A, Asiegbu EC, Nanven MB, Lawal-Atolagbe T, Lawal-Lah F, Awoyale OD, Abubakar AT, Sahabi K, Babandi ZS (2023). A cross-sectional survey of public knowledge of the monkeypox disease in Nigeria. BMC Public Health.

[57] Kumar N, Ahmed F, Raza MS, Rajpoot PL, Rehman W, Khatri SA, Mohammed M, Muhammad S, Ahmad R (2022). Monkeypox Cross-Sectional Survey of Knowledge, Attitudes, Practices, and Willingness to Vaccinate among University Students in Pakistan. Vaccines (Basel).

[58] Rodrigues CMC, Plotkin SA (2020). Impact of Vaccines; Health, Economic and Social Perspectives. Front Microbiol.

[59] Manirambona E, Musa SS, Shomuyiwa DO, Salam FA, John OO, Dinyo DGA, Haruna UA, Sow AU, Lucero-Prisno DE, Ezie KN, Samai M, Aziato L (2022). The monkeypox virus: A public health challenge threatening Africa. Public Health Chall.

[60] Ajibo HT, Obi-keguna C, Ugwuoke J (Dec). Monkey pox and destabilization of community life in Nigeria: Implication for social work practice. JIOSR Journal Of Humanities And Social Science.

[61] Abbas S, Karam S, Schmidt-Sane M, Palmer J (2022). Social considerations for monkeypox response.

[62] Tambo E, Al-Nazawi AM (2022). Combating the global spread of poverty-related Monkeypox outbreaks and beyond. Infect Dis Poverty.

